# The association between coach-athlete relationship and self-reported task, contextual, and overall athletic performance among university student-athletes

**DOI:** 10.3389/fpsyg.2026.1879798

**Published:** 2026-09-29

**Authors:** Mehdi Duyan, İlker Günel, Mehmet Bayansalduz

**Affiliations:** 1 Faculty of Sports Sciences, Inonu University, Malatya, Turkiye; 2 School of Physical Education and Sports, Osmaniye Korkut Ata University, Osmaniye, Turkiye; 3 Faculty of Sports Sciences, Topkapi University, Istanbul, Turkiye

**Keywords:** athletic performance, coach-athlete relationship, contextual performance, hierarchical regression analysis, task performance

## Abstract

**Introduction:**

The coach-athlete relationship is a fundamental psychosocial factor associated with athlete performance. However, its associations with self-reported task performance, contextual performance, and overall athletic performance have not been sufficiently examined within a single model. Drawing on theoretical frameworks such as the 3C model and Self-Determination Theory, this study aimed to investigate the associations between the coach-athlete relationship and two performance sub-dimensions (task performance and contextual performance) and their composite overall score (athletic performance), with overall athletic performance operationalized as a composite score reflecting athletes' global self-perceived performance.

**Method:**

This quantitative study employed a correlational survey design with 276 university student-athletes (154 male and 122 female) competing in individual and team sports. The Coach-Athlete Relationship Scale and the Athletic Performance Scale (comprising task performance and contextual performance sub-dimensions and an overall composite score) were used. Hierarchical regression analysis was conducted to examine the statistical association of the coach-athlete relationship with self-reported performance dimensions after controlling for demographic variables.

**Results:**

The coach-athlete relationship was positively and significantly correlated with task performance *(r* = *0.412, p*<*0.001)*, contextual performance *(r* = *0.465, p*<*0.001)*, and overall athletic performance *(r* = *0.461, p*<*0.001)*. Hierarchical regression analysis showed that the coach-athlete relationship demonstrated a significant statistical association with task performance *(*β = *0.514, p*<*0.001)*, contextual performance *(*β = *0.563, p*<*0.001)*, and overall athletic performance (β = *0.560, p*<*0.001)*, after controlling for demographic variables.

**Conclusion:**

A high-quality coach-athlete relationship is positively associated with athletes' self-reported task performance, contextual performance, and overall athletic performance. These findings suggest that the coach-athlete relationship may be important in relation to athletes' social, cooperative, and team-oriented behaviors. However, as all measures were self-reported, the results reflect perceived rather than objective performance.

## 1 Introduction

The coach-athlete relationship (CAR) is considered one of the most fundamental interpersonal dynamics associated with an athlete's success, motivation, and performance (Jowett, 2007). According to the 3C model developed by Jowett and Ntoumanis (2004), which defines the CAR through the dimensions of Closeness, Commitment, and Complementarity, high-quality relationships are associated with an environment of trust, respect, and mutual support for the athlete, which, according to the 3C model, is theorized to be associated with optimal performance outcomes. This proposition has received empirical support in studies examining the relationship between relationship quality and athlete satisfaction, motivation, and perceived performance (Adie and Jowett, 2010; Rhind et al., 2012; Felton and Jowett, 2013).

Existing research has examined performance-related outcomes associated with the CAR, drawing on diverse types of evidence. Psychological evidence includes studies examining athletes' motivation, satisfaction, perceived competence, and team cohesion (Wylleman, 2000; Poczwardowski et al., 2002; Jowett and Chaundy, 2004). Self-reported performance evidence includes athletes' subjective evaluations of their own performance, as measured in studies on task and contextual performance (Akbiyikli and Sürgevil Dalkiliç, 2020; Duyan, 2021; Bora, 2025) and the relationship between coach leadership behavior and athlete performance (Liu et al., 2025). Objective sport performance evidence includes direct measures of physical and cognitive performance; Davis et al. (2018) incorporated objective physical performance (5-meter shuttle test), cognitive performance (Stroop test), and physiological stress response (salivary cortisol), while Phillips et al. (2023) demonstrated that CAR quality was associated with objective batting performance in elite cricket using data from 28,215 balls faced, and Phillips et al. (2026) extended this to bowling performance using 50,159 deliveries, identifying communication strategies, psychological safety, and collective efficacy as factors in this relationship. Leadership-related evidence has examined how coach behaviors such as transformational leadership relate to athlete performance outcomes (Ardern et al., 2013; Bormann et al., 2016; Karayel et al., 2024; Clare et al., 2025).

Athletic performance (AP) is a multidimensional phenomenon that includes not only physical skill and tactical coordination but also psychological and social factors such as motivation, communication, team cooperation, and psychological resilience (Filho et al., 2015). In the sports literature, AP is conceptualized as a holistic construct that reflects an athlete's overall ability to meet the demands of the sport environment (Jowett and Slade, 2021; López de Subijana et al., 2024). The distinction between “task performance” and “contextual performance,” frequently used in organizational psychology literature, remains valid in the sports context as well (Chen et al., 2011; Motowidlo et al., 2014). While task performance (TP) refers to an athlete's technical-tactical competence, physical preparation, and ability to fulfill in-game responsibilities (Cormack et al., 2008; Baker and Young, 2014); contextual performance (CP) encompasses social and psychological processes that are associated with performance, such as team cohesion (Felton et al., 2021), leadership (Chelladurai, 2007), psychological support (Freeman and Rees, 2010), communication (Davis et al., 2018; Kuruzović and Ðorić, 2023), and long-term athlete development (Isoard-Gautheur et al., 2016; Gustafsson et al., 2017).

Akbiyikli and Sürgevil Dalkiliç (2020), in their study on professional athletes, found that leader-member exchange was positively associated with TP and CP, as measured using the Individual Performance Scale, which relies on athlete self-report scores. Similarly, Duyan (2021), in a study on combat sports athletes, concluded that a high-quality CAR was positively related to AP. Pan and Sui (2025) found that CAR had a significant positive association with team performance *(*β = *0.556, p*<*0.001)* among 1,566 Chinese athletes, with emotional intelligence and athletic engagement as factors in this relationship. Zhang and Rhim (2024) demonstrated that CAR had a positive association with motor behavior among Chinese college athletes, with psychological needs as a factor in this relationship. Yuan (2024) found that male college athletes reported more positive CAR perceptions than their female counterparts. Kaygusuz et al. (2021), in a qualitative study investigating optimal psychological performance profiles of football players, indicated that coach behaviors were related to players' optimal performance.

It is important to note that the literature on CAR and performance encompasses studies using diverse outcome measures. While some early studies employed objective performance indicators such as competition results or coach evaluations (Cormack et al., 2008; Baker and Young, 2014), recent research has added to this evidence base. Complementing this, a meta-analysis by Zhu et al. (2024) found a moderate positive association between coach leadership behaviors and athlete satisfaction and cohesion. Similarly, a recent systematic review by Kuruzović and Ðorić (2023) reported that positive CAR are associated with performance, motivation, and protection against stress and burnout. However, the majority of studies have relied on self-report measures of perceived performance (Roux and Trouilloud, 2021; Cruz, 2025). The present study adopts the latter approach, examining athletes' self-reported perceptions of their performance.

Beyond performance outcomes, CAR has also been linked to other important athlete variables. For instance, Gencer and Öztürk (2018) found that CAR was significantly associated with sport-confidence among Turkish national wrestlers, while Güllü (2018) reported that CAR was positively correlated with sportsmanship orientation among Turkish university student-athletes. Furthermore, Jowett et al. (2017) found that athletes' perceptions of CAR (specifically closeness and commitment) were significantly associated with a range of positive coach behaviors, including physical training planning, technical skill development, and competition strategies.

Although the CAR in the current literature is generally associated with overall self-reported performance outcomes, as systematically reviewed by Roux and Trouilloud (2021), previous studies have largely focused on specific populations. For example, Duyan (2021) examined CAR among combat athletes, Yuan (2024) investigated CAR in team sports, and Zhang and Rhim (2024) studied CAR among Chinese college athletes. However, the associations of CAR with TP, CP, and their composite overall score (AP) have not been examined together within a single study in the context of Turkish university student-athletes. Therefore, this study aims to fill this gap by investigating the associations between CAR and these two performance sub-dimensions and their composite overall score among Turkish university student-athletes. This simultaneous examination is theoretically significant because it allows us to determine whether CAR's associations differ across performance dimensions, particularly whether CAR is differentially associated with contextual performance (social and cooperative behaviors) vs. task performance (technical-tactical competence). Furthermore, by operationalizing AP as a composite score, we provide a holistic indicator of athletes' self-perceived performance while recognizing that performance is a multidimensional construct. This approach advances current understanding by clarifying the associations of CAR with different performance dimensions and by testing these relationships in a previously understudied cultural context.

Rather than discovering a new relationship, the primary contribution of this study lies in differentiating how CAR quality relates to distinct performance-related perceptions—task performance, contextual performance, and their composite overall score—within a single model. Specifically, while previous research has largely examined CAR in relation to either overall performance or specific performance indicators in isolation, the present study offers a more nuanced understanding by simultaneously assessing CAR‘s associations with two performance sub-dimensions (TP and CP) and their composite overall score (AP). This study contributes to the literature by simultaneously examining CAR‘s associations with task performance, contextual performance, and their composite overall score (AP) within the Turkish university student-athlete context. Such differentiation is theoretically meaningful because it clarifies the relative importance of CAR for different aspects of athlete functioning and offers more targeted implications for coaching practice. The conceptual framework and detailed definitions of each construct, along with the theoretical rationale for the proposed hypotheses, are presented in the following section.

### 1.1 Coach-Athlete Relationship (CAR)

The CAR is defined as a unique interpersonal dynamic in which emotions, thoughts, and behaviors between a coach and an athlete are mutually interconnected (Jowett and Ntoumanis, 2004). This relationship is characterized by mutual trust, respect, commitment, and cooperation, which collectively are associated with a supportive environment conducive to athlete development and success (Jowett, 2007). According to the 3C model, the CAR comprises three dimensions: Closeness (emotional tone, trust, respect, and appreciation), Commitment (intention to maintain the relationship over time, dedication, and shared goals), and Complementarity (cooperative interactions, role clarity, and mutual support during training and competition) (Jowett, 2007). These three sub-dimensions are interrelated and collectively determine the overall quality of the CAR. When these three sub-dimensions are present, the relationship is considered optimal and is associated with positive outcomes for both the athlete and the coach (Jowett and Ntoumanis, 2004). A high-quality CAR is associated with an environment where athletes feel supported, valued, and motivated, which in turn is associated with their engagement and performance outcome (Mageau and Vallerand, 2003). Conversely, a low-quality CAR characterized by a lack of closeness, commitment, or complementarity may be associated with negative outcomes such as decreased motivation, increased burnout, and poor performance (Yildiz, 2011; Isoard-Gautheur et al., 2016). Moreover, Jowett et al. (2017) provided empirical evidence that athletes' perceptions of closeness and commitment are associated with positive coach behaviors (e.g., technical skill instruction, competition strategies, mental preparation), while complementarity was primarily associated with positive personal rapport.

### 1.2 Task Performance (TP)

TP is a concept originally developed in organizational psychology to describe the core technical activities and responsibilities that are related to organizational goals (Motowidlo et al., 1997; Goodman and Svyantek, 1999). In the sport context, TP refers to an athlete‘s technical-tactical competence, physical preparation, and ability to fulfill in-game responsibilities (Filho et al., 2015; Akbiyikli and Sürgevil Dalkiliç, 2020). It encompasses the core, technical aspects of sport performance that are relevant to achieving competitive goals, such as executing specific skills (e.g., shooting, passing, tackling), following tactical instructions, and meeting physical demands (e.g., speed, strength, endurance) (Mageau and Vallerand, 2003). TP is often measured through objective indicators such as game statistics, coach ratings, or performance tests, and is considered an important outcome of most sport training programs (Baker and Young, 2014). A positive CAR is positively associated with TP, which is related to athletes' openness to learning, effort, and competence perception. When athletes trust their coaches, they are more receptive to feedback, more willing to try new techniques, and more persistent in the face of challenges (Davis et al., 2022). Through the trust an athlete places in their coach, they can learn challenging technical instructions and demonstrate effort to follow training directives (Hampson and Jowett, 2012). This process is associated with technical learning and skill development, thereby being associated with TP. Moreover, a supportive CAR is associated with reduced anxiety and fear of failure, which is associated with athletes' ability to focus their cognitive resources on skill execution rather than on worries about evaluation or criticism (Sagar and Jowett, 2015).

### 1.3 Contextual Performance (CP)

CP encompasses social and psychological processes that are associated with performance, such as team cohesion (Felton et al., 2021), leadership (2007), psychological support (Freeman and Rees, 2010), communication (Davis et al., 2018; Kuruzović and Ðorić, 2023), and long-term athlete development (Isoard-Gautheur et al., 2016; Gustafsson et al., 2017). Originally conceptualized in organizational psychology (Motowidlo et al., 2014), CP has been adapted to the sports context to describe behaviors that support the team‘s social and psychological functioning. CP includes behaviors such as cooperation, dedication, social support, and extra effort shown by an athlete within the team (Wang and Tong, 2024). Examples of CP in sports include helping a teammate who is struggling, encouraging others after a mistake, volunteering for extra duties, and maintaining a positive attitude even during difficult times (Wang and Tong, 2024). These behaviors are not directly related to technical execution but establish a positive team environment that is associated with overall effectiveness. In team sports, CP is important because it is associated with group cohesion, and is associated with reduced interpersonal conflict, and is associated with collective efficacy (Filho et al., 2015). According to Self-Determination Theory (Ryan and Deci, 2000), the CAR is associated with intrinsic motivation, which is related to the satisfaction of athletes' needs for autonomy, competence, and relatedness, and is associated with CP (Mageau and Vallerand, 2003). When athletes feel that their coach respects their autonomy, acknowledges their competence, and cares about them as individuals, they internalize team values and demonstrate prosocial behaviors (Jowett, 2017). High-quality CAR is associated with a sense of belonging, motivation, psychological well being, and social bonds, all of which are associated with CP. Furthermore, a positive CAR is associated with prosocial behavior, which is reflected in athletes' supportive and respectful conduct (Bolter and Weiss, 2013). Jowett and Chaundy (2004) found that CAR was significantly associated with team cohesion, with athletes' commitment to their coach being significantly associated with task cohesion *(*β = *0.32)*, while assumed similarity between athletes' self-perceptions and meta-perceptions of the relationship was associated with social cohesion.

### 1.4 Athletic Performance (AP)

Athletic performance (AP) is conceptualized as a multidimensional construct encompassing physical, technical, cognitive, and psychological factors that are associated with an athlete‘s overall effectiveness in sport (Jowett and Slade, 2021; López de Subijana et al., 2024). AP integrates both task and contextual dimensions, recognizing that successful performance is associated with not only technical-tactical skills but also social and psychological factors such as motivation, communication, team cooperation, and psychological resilience (Filho et al., 2015). In this study, AP represents a higher-order composite indicator that integrates task performance (TP) and contextual performance (CP). While TP focuses on the technical and tactical execution of sport-specific tasks, and CP reflects athletes' social and cooperative behaviors within the team, AP offers a comprehensive assessment of an athlete‘s overall contribution. This hierarchical structure, in which TP and CP serve as sub-dimensions of an overarching AP construct, is consistent with the organizational psychology literature, where task and contextual performance are often combined to form an overall performance evaluation (Motowidlo et al., 2014). In the present study, AP is operationalized as the composite score of the self-reported task and contextual performance items, reflecting athletes' global perception of their performance rather than objective performance outcomes (Filho et al., 2015; Akbiyikli and Sürgevil Dalkiliç, 2020). AP is often assessed through coach ratings, self-reports, or a combination of objective and subjective indicators (Jowett and Slade, 2021). A positive CAR is associated with AP through its association with technical and tactical development and through psychological factors such as motivation, trust, and psychological safety (Jowett, 2017). One aspect involves coaches offering high-quality instruction, feedback, and training programs that are associated with athletes' skills and physical capacities. Another aspect involves coaches establishing a supportive environment that is associated with athletes' confidence, is associated with reduced stress, and is associated with intrinsic motivation (Davis et al., 2018). Given that AP encompasses both TP and CP, it is expected that CAR will be positively associated with AP through its associations with both technical skill development and prosocial team behaviors. Pan and Sui (2025) demonstrated that CAR had a positive association with team performance *(*β = *0.556, p*<*0.001*) among a large sample of Chinese athletes, with emotional intelligence and athletic engagement as factors in this relationship.

### 1.5 Hypothesized relationships between CAR and performance dimensions

A high-quality CAR is expected to be positively associated with TP, CP, and AP. The theoretical and empirical rationale for these associations is grounded in the 3C model (Ntoumanis et al., 2004), Self-Determination Theory (Ryan and Deci, 2000), and previous empirical findings. Specifically, Akbiyikli and Sürgevil Dalkiliç (2020) found positive associations between leader-member exchange (LMX) and self-reported TP and CP among professional athletes, which is consistent with the role of interpersonal relationships in performance. Duyan (2021) reported a positive association between CAR and self-reported AP among combat athletes. Zhang and Rhim (2024) demonstrated that CAR had a positive association with self-reported motor behavior among Chinese college athletes, with psychological needs as a factor in this relationship. More recently, Jowett (2024) emphasized that high-quality CAR, as defined by the 3C model, are associated with performance-related outcomes, with empirical evidence indicating that athletes who report high-quality relationships with their coaches also report more favorable performance-related outcomes and competence development. This body of evidence, based on self-report measures, is consistent with the positive association between CAR quality and various performance dimensions. Pan and Sui (2025) found that CAR was positively associated with team performance, as measured using athlete-rated questionnaire items, with emotional intelligence and athletic engagement as factors in this relationship.

#### 1.5.1 Coach-athlete relationship and task performance (H1)

A positive CAR is positively associated with athletes' openness to learning, effort, and competence perception. When athletes perceive high levels of closeness, commitment, and complementarity with their coaches, they trust their coaches' expertise and guidance (Hampson and Jowett, 2012). Trust in the coach may be related to athletes' assimilation of technical instructions and feedback, which is associated with technical-tactical skills and physical preparation (Mageau and Vallerand, 2003; Hampson and Jowett, 2012). Furthermore, a supportive CAR is associated with reduced performance anxiety, which is associated with athletes' ability to focus on skill execution (Sagar and Jowett, 2015). Therefore, it is hypothesized that:

*H*_1_*: CAR is positively associated with TP*.

#### 1.5.2 Coach-athlete relationship and contextual performance (H2)

According to Self-Determination Theory, a high-quality CAR is theorized to be related to the satisfaction of athletes' basic psychological needs (autonomy, competence, and relatedness), which may be related to intrinsic motivation and prosocial behaviors such as cooperation, dedication, and social support within the team (Ryan and Deci, 2000; Mageau and Vallerand, 2003). When athletes feel that their coach respects their input (autonomy), believes in their abilities (competence), and cares about them personally (relatedness), they internalize team goals and engage in behaviors that are associated with the collective (Jowett, 2017). Athletes in positive CAR environments exhibit contextual behaviors that are associated with team functioning. Moreover, coaches who model respect, fairness, and supportiveness are associated with athletes' supportive behaviors, which are associated with CP (Bolter and Weiss, 2013). Jowett and Chaundy (2004) found that CAR was significantly associated with team cohesion, a component of CP. Therefore, it is hypothesized that:

*H*_2_*: CAR is positively associated with CP*.

#### 1.5.3 Coach-athlete relationship and athletic performance (H3)

AP is expected to be positively associated with CAR in relation to technical and tactical development and psychological factors such as motivation, trust, and psychological safety, which have also been associated with overall AP (Jowett, 2017). Because AP encompasses both TP and CP, the positive associations of CAR with each of these sub-dimensions are expected to be associated with the overall association between CAR and AP (Jowett and Slade, 2021). Pan and Sui (2025) found support for this hypothesis, reporting an association between CAR and team performance *(*β = *0.556)* as well as associations through emotional intelligence and athletic engagement. Therefore, it is hypothesized that:

*H*_3_*: CAR is positively associated with AP*.

## 2 Materials and methods

### 2.1 Research model

This study is a quantitative investigation based on a correlational survey model, aiming to investigate the associations between the CAR and AP, comprising two sub-dimensions (TP and CP) and their composite overall score (AP). AP in this study refers to self-reported TP and CP scale scores. This scale comprises two sub-dimensions: TP (9 items) and CP (7 items), with the overall composite score representing athletes' global self-perceived AP. The correlational survey model is a research design intended to determine the level and direction of the relationship between two or more variables (Karasar, 2024). Hierarchical regression analysis was utilized to test the relationships among the variables. The research model is illustrated in Figure 1.

**Figure 1:**
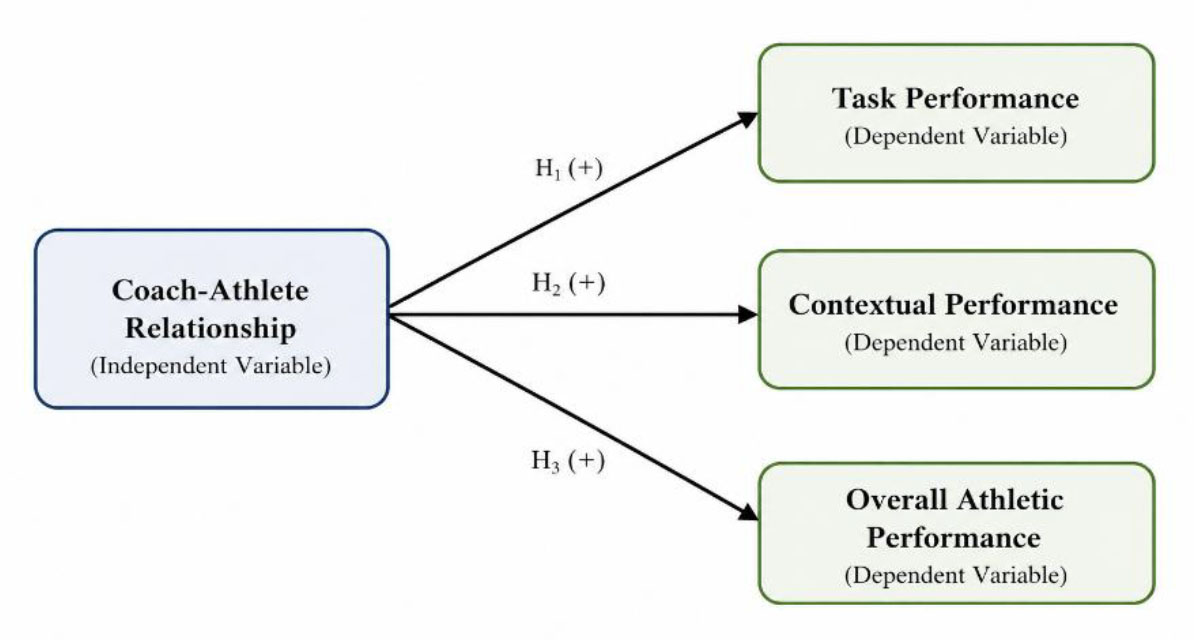
Research model.

### 2.2 Population and sample

The population of this study consists of licensed student-athletes actively competing in individual and team sports in Türkiye. The sample of the study comprises a total of 276 university student-athletes, 154 male and 122 female, studying at various universities in the Eastern Anatolia Region of Türkiye, selected via convenience sampling. The following inclusion criteria were established for participant selection: (a) being 18 years of age or older, (b) having been regularly training for at least 1 year, and (c) interacting with their coach at least twice a week. Exclusion criteria included: (a) having experienced a serious sports injury within the last 6 months, (b) not being a member of a university sports team, and (c) not currently pursuing an active sports career.

Initially, 347 online survey links were sent via athletes and their coaches to collect data from university student-athletes competing in individual and team sports. Responses were received from 283 athletes (81.6%). The data were processed in an Excel file and then transferred to Jamovi 2.7.6 statistical program. Subsequently, normality assumptions of the data were examined. Seven extreme outliers were removed from the analysis, and the remaining 276 data points were used for further analysis.

Various opinions exist in the literature regarding adequate sample size. For instance, Valdivia and Dai (2024) suggest that a sample size of 250 or more is sufficient. The final sample of 276 athletes in this study meets this recommendation (*N* > 270). Kim et al. (2015) propose the following guidelines for absolute sample size: (a) *N* > 200 as a large sample, (b) *N* ≈ 150 as a medium sample, and (c) *N* < 100 as a small sample. Tabachnick and Fidell (2001) argue that a minimum of 200 participants is necessary for hierarchical regression analysis, and a larger sample is preferable. Thus, the sample size (*N* = 276) was considered adequate for the analyses, exceeding the recommended minimum of 200 participants (Tabachnick and Fidell, 2001; Kim et al., 2015).

### 2.3 Self-report measures

#### 2.3.1 Coach–Athlete Relationship (CAR)

To measure the coach–athlete relationship, the athlete form of the scale developed by Jowett and Ntoumanis (2004) and adapted into Turkish by Altintaş et al. (2012) was used. The scale consists of three sub-dimensions (Closeness, Commitment, and Complementarity) and a total of 11 items. Items were rated on a 7-point Likert-type scale ranging from (1-Strongly disagree) to (7-Strongly agree). In the adaptation study, Cronbach's alpha reliability coefficients for the athlete form were reported as 0.90, 0.90, and 0.82, respectively (Altintaş et al., 2012).

#### 2.3.2 Athletic Performance (AP)

To assess athlete performance, a 16-item scale measuring Task Performance and Contextual Performance dimensions was employed. The task-performance sub-dimension comprises 9 items adapted from the work of Goodman and Svyantek (1999), while the contextual-performance sub-dimension includes 7 items adapted from Jawahar and Carr (2007). The Turkish adaptation of the scale was carried out by Sahin (2018) and Bagci (2014). Items were answered on a 5-point Likert-type scale ranging from (1-Strongly disagree) to (5-Strongly agree). For the entire scale, the Cronbach's alpha reliability coefficient was 0.90; for the task-performance and contextual-performance sub-dimensions, the values were 0.90 and 0.90, respectively (Akbiyikli, 2019). Overall athletic performance (AP) was operationalized as the composite mean score of all 16 items, representing athletes' global self-perceived performance. Thus, AP reflects a holistic composite indicator rather than an independent third dimension.

### 2.4 Data collection

Prior to commencing the data collection phase of the study, ethical approval was obtained from the Inönü University Social and Human Sciences Research Ethics Committee under protocol number 2025-23/21. Data collection took place between January and March 2026, following the approval of the ethics committee. The research was conducted in accordance with the ethical principles outlined in the Declaration of Helsinki. Data were collected using an online survey method. Contact was established with the coaches and athletes of the relevant universities, and the online survey link was shared with them to be distributed to potential participants. Potential participants were informed that the survey would take approximately 10–15 min to complete, and that their responses would remain anonymous and confidential. The introductory page of the survey clearly stated the purpose of the study, the voluntary nature of participation, data confidentiality, and contact information of the researcher. The survey link was sent to a total of 300 athletes, and valid responses were received from 283 participants, resulting in a response rate of 94.3%. During data preparation for analysis, the data of 7 participants identified as having extreme values (outliers) were removed. Consequently, the final analyses were conducted using data from 276 athletes.

### 2.5 Data analysis

The data of the study were analyzed using Jamovi 2.7.6 statistical package program. Subsequently, the skewness and kurtosis values of the remaining data were examined to test the normality assumption. Since these values fell within the range of −1.5 to +1.5, it was concluded that the data were normally distributed (Tabachnick et al., 2013). Descriptive statistics (mean, standard deviation) were calculated for the variables, and the relationship between variables was examined using Pearson correlation analysis. The relationship between the independent variable (CAR) and the dependent variables (task performance, contextual performance, and athletic performance) was investigated using hierarchical regression analysis.

Confirmatory Factor Analysis (CFA) was applied to evaluate the construct validity and reliability of the scales. Model fit was assessed using the χ^2^/df ratio, RMSEA, CFI, TLI, and SRMR indices. For reliability analysis, Cronbach‘s Alpha (α) and McDonald‘s Omega (ω) coefficients were computed, along with Average Variance Extracted (AVE) and Composite Reliability (CR) values for convergent validity. To test the hypothesized relationships proposed in the research model, hierarchical regression analysis was conducted. The statistical significance level was set at *p* < 0.05 for all analyses.

## 3 Results

### 3.1 Descriptive statistics

The population of the study consists of university athletes engaged in individual and team sports, while the sample comprises 276 university athletes studying at various universities in Eastern Anatolia, Türkiye. Of the participants, 55.8% (*n* = 154) are male and 44.2% (*n* = 122) are female. In terms of age distribution, 49.3% of the participants are 25 years old or younger, 47.5% are between 26–30 years old, and 3.3% are 31 years old or above. Regarding sport type, 57.2% participate in individual sports, while 42.8% are involved in team sports. An examination of the distribution of sports disciplines within the sample reveals that among team sports, basketball holds the highest proportion at 20.7%, whereas among individual sports, Muaythai constitutes the highest proportion at 15.9%. In terms of total sports experience, 56.1% of the athletes have 5 or more years of sports background. The proportion of national athletes within the sample is 8.0%. Detailed information regarding the demographic and athletic characteristics of the sample is presented in Table 1.

**Table 1:** Demographic characteristics of the athletes.

***Variables***		*f*	%
Gender	Male	154	55.8
	Female	122	44.2
	Total	276	100.0
Marital status	Married	34	12.3
	Single	242	87.7
	Total	276	100.0
Age	25 years and under	136	49.3
	26–30 years	131	47.5
	31 years and above	9	3.3
	Total	276	100.0
Type of sport	Individual Sports	158	57.2
	Team Sports	118	42.8
	Total	276	100.0
Sports discipline	Taekwondo	36	13.0
	Muaythai	44	15.9
	Volleyball	29	10.5
	Basketball	57	20.7
	Soccer	35	12.7
	Table Tennis	12	4.3
	Swimming	21	7.6
	Wrestling	26	9.4
	Judo	16	5.8
	Total	276	100.0
Total sports experience	1–2 years	24	8.7
	3–4 years	42	15.2
	5–6 years	79	28.6
	7–8 years	56	20.3
	9 years and above	75	27.2
	Total	276	100
National athlete	Yes	22	8.0
	No	254	92.0
	Total	276	100.0

### 3.2 Common method bias

To assess common method bias, Harman‘s single-factor test was conducted. The unrotated factor solution explained 31.6% of the total variance, which is below the recommended threshold of 50% (Podsakoff et al., 2003). While this provides some reassurance, it should be noted that Harman‘s single-factor test offers only a limited diagnostic indication and does not fully rule out common method variance.

### 3.3 Confirmatory factor and reliability analysis

To evaluate the validity of the measurement model and confirm the theoretical structure, Confirmatory Factor Analysis (CFA) was conducted. The analyses were performed using Jamovi 2.7.6 software. Based on modification indices, error covariances were added between specific item pairs: CAR1–CAR2 and CAR7–CAR8 for the CAR scale; items 1–2 for the TP scale; items 8–11 for the CP scale; and items 1–2 for the AP scale. The fit indices for each scale are presented in Table 2. All scales demonstrated acceptable to excellent model fit, with CFI values ranging from 0.983 to 0.992, TLI values from 0.980 to 0.990, RMSEA values from 0.05 to 0.08, and SRMR values from 0.05 to 0.07. The fit indices for each scale are presented in Table 2 (see notes for abbreviations and modification details). As shown in Table 2, all scales demonstrated acceptable to excellent model fit.

**Table 2:** Confirmatory factor analysis fit indices.

Scale	χ^2^/df	CFI	TLI (NNFI)	NFI	RMSEA	SRMR	Result
**CAR**	135/42	0.992	0.990	0.989	0.08	0.07	**Acceptable**
**TP**	65.0/26	0.990	0.986	0.986	0.075	0.065	**Acceptable**
**CP**	24.5/13	0.991	0.985	0.980	0.05	0.05	**Acceptable**
**AP**	250/103	0.983	0.980	0.972	0.07	0.07	**Acceptable**

χ^2^/df, Chi-square/degrees of freedom ratio; CFI, Comparative Fit Index; TLI (NNFI), Tucker-Lewis Index (Non-Normed Fit Index); NFI, Normed Fit Index; RMSEA, Root Mean Square Error of Approximation; SRMR, Standardized Root Mean Square Residual; CAR, Coach-Athlete Relationship; TP, Task Performance; CP, Contextual Performance; AP, Athletic Performance. Acceptability thresholds: CFI, TLI, NFI ≥ 0.95 for excellent fit, ≥ 0.90 for acceptable fit; RMSEA and SRMR ≤ 0.05 for excellent fit, ≤ 0.08 for acceptable fit (Hu and Bentler, 1999; Gürbüz, 2019).

### 3.4 Findings of reliability and convergent validity

After the confirmatory factor analysis of our measurement model, the validity of the structural equation model was ensured through a comprehensive evaluation using Reliability and Convergent Validity analyses. Reliability analyses were conducted using both Cronbach‘s Alpha (α) as an indicator of internal consistency and Composite Reliability (ω), which accounts for measurement error more comprehensively. Convergent validity was tested using the Average Variance Extracted (AVE) value, which indicates the extent to which each latent variable is explained by its assigned indicators. Contemporary psychometrics recommends the combined use of ω and AVE for reliability and convergent validity assessments, due to the limitations of Cronbach‘s Alpha (Ringle and Sarstedt, 2021).

According to the results presented in Table 3, the CAR scale demonstrates excellent reliability (α = 0.896, ω = 0.921) and convergent validity *(AVE* = *0.663* > *0.50)*. The TP sub-dimension exhibited very good reliability *(*α = *0.840*, ω = *0.857)* and met the sufficient convergent validity criterion *(AVE* = *0.540* > *0.50)*. For the CP sub-dimension, reliability coefficients were found to be acceptable *(*α = *0.767*, ω = *0.795);* however, the AVE value *(0.482)* was observed to be slightly below the ideal threshold of 0.50. Nevertheless, given that the reliability coefficients and CR values for the CP sub-dimension are at an acceptable level, it was retained in the SEM model. Consequently, the measurement model was found to possess values ranging from acceptable to excellent.

**Table 3:** Internal consistency and convergent validity indices of the scales.

**Variable**	Cronbach's α	Ordinal α	Composite reliability (ω1/ω_2_)	Hierarchical ω (ω3)	AVE
**CAR**	0.883	0.941	0.877	0.900	0.609
**TP**	0.845	0.897	0.836	0.851	0.528
**CP**	0.740	0.846	0.728	0.737	0.519
**AP**	0.886	0.930	0.893	0.923	0.506

α, Cronbach's Alpha; ω, McDonald's Omega; AVE, Average Variance Extracted; CR, Composite Reliability. Acceptability thresholds: α & ω > 0.70, AVE > 0.50 (Gürbüz and Sahin, 2014; Gürbüz, 2019; Ringle and Sarstedt, 2021).

### 3.5 Correlation analysis

Descriptive statistics and correlation analysis results for the variables are presented in Table 4. The means for CAR *(M* = *4.59, SD* = *0.43)*, TP *(M* = *4.38, SD* = *0.47)*, CP *(M* = *4.52, SD* = *0.43)*, and AP *(M* = *4.47, SD* = *0.39)* were at a high level. CAR was positively and significantly correlated with TP *(r* = *0.412, p*<*0.001)*, CP *(r* = *0.465, p*<*0.001)*, and AP *(r* = *0.461, p*<*0.001)*.

**Table 4:** Means, standard deviations, and correlations of the variables.

**Variables**	M	SD	1	2	3	4
**1.CAR**	4.59	0.43	1			
**2.TP**	4.38	0.47	0.412^***^	1		
**3.CP**	4.52	0.43	0.465^***^	0.731^***^	1	
**4.AP**	4.47	0.39	0.461^***^	0.854^***^	0.894^***^	1

*X*, Mean; *SD*, Standard Deviation; CAR, Coach-Athlete Relationship; TP, Task Performance; CP, Contextual Performance; AP, Athletic Performance. ^***^ *p* < 0.001.

### 3.6 Hierarchical regression

Hierarchical regression analysis showed that demographic variables had no significant association with CP. Gender was significantly associated with TP *(*β = −*0.134, p* = *0.013)* and AP *(*β = −*0.126, p* = *0.017)*, but not for CP *(*β = −*0.102, p* = *0.055)*. When CAR was added in Step 2, it showed a significant association with TP, CP, and overall AP *(p*<*0.001)*, explaining an additional 26.3% of the variance in TP, 31.6% in CP, and 31.3% in AP. CAR was associated with CP *(*β = *0.563)*, AP *(*β = *0.560)*, and TP *(*β = *0.514)*. The final models explained 29.8%, 31.8%, and 33.4% of the variance in TP, CP, and AP, respectively (see Tables 5, 6, 7).

**Table 5:** Hierarchical regression analysis results for task performance.

**Independent variables**	Step 1			Step 2		
	Beta	t	p	Beta	t	p
1. Gender	−0.147	−2.330	0.021	−0.134	−2.502	0.013
2. Age	−0.109	−1.539	0.125	−0.118	−1.949	0.052
3. Marital status	0.066	1.034	0.302	0.089	1.640	0.102
4. Sport type	−0.054	−0.884	0.377	−0.013	−0.246	0.806
5. Sport branch	−0.037	−0.553	0.581	−0.077	−1.353	0.177
6. Education level	0.106	1.436	0.152	0.057	0.904	0.367
7. Total sport experience	0.172	2.003	0.046	0.170	2.329	0.021
8. Current branch experience	−0.107	−1.059	0.291	−0.083	−0.964	0.336
9. Duration with current coach	0.039	0.465	0.642	−0.012	−0.170	0.865
10. National athlete	−0.147	−2.330	0.021	0.040	0.768	0.443
**11. Coach-Athlete Relationship (CAR)**	-	-	-	**0.514**	10.100	**<0.001**
F		2.345			4.780	
R^2^		0.171			0.313	
Adjusted R^2^		0.035			0.298	

Standardized beta coefficients are used. Model (*p*) < 0.001; Tolerance: 0.970; VIF: 1.031; Durbin Watson: 1.925.

**Table 6:** Hierarchical regression analysis results for contextual performance.

**Independent variables**	Step 1			Step 2		
	Beta	t	p	Beta	t	p
1. Gender	−0.116	−1.804	0.072	−0.102	−1.926	0.055
2. Age	−0.118	−1.636	0.103	−0.128	−2.139	0.033
3. Marital status	0.044	0.690	0.490	0.070	1.309	0.191
4. Sport type	−0.003	−0.053	0.957	0.042	0.810	0.418
5. Sport branch	−0.044	−0.642	0.522	−0.088	−1.557	0.121
6. Education level	0.096	1.282	0.201	0.043	0.687	0.493
7. Total sport experience	0.027	0.313	0.754	0.026	0.356	0.722
8. Current branch experience	−0.038	−0.370	0.712	−0.012	−0.140	0.889
9. Duration with current coach	0.077	0.909	0.364	0.021	0.304	0.761
10. National athlete	0.064	1.017	0.310	0.032	0.613	0.540
**11. Coach-Athlete Relationship (CAR)**	-	-	-	**0.563**	**11.206**	**<0.001**
F		1.051			12.814	
R^2^		0.038			0.345	
Adjusted R^2^		0.002			0.318	

Standardized beta coefficients are used. Model (*p*) < 0.001; Tolerance = 0.970; VIF = 1.031; Durbin Watson = 1.924.

**Table 7:** Hierarchical regression analysis results for athletic performance.

**Independent variables**	Step 1			Step 2		
	Beta	t	p	Beta	t	p
1. Gender	−0.140	−2.201	0.029	−0.126	−2.411	0.017
2. Age	−0.118	−1.656	0.099	−0.128	−2.169	0.031
3. Marital status	0.059	0.927	0.355	0.084	1.602	0.110
4. Sport type	−0.034	−0.549	0.584	0.011	0.219	0.827
5. Sport branch	−0.042	−0.619	0.536	−0.086	−1.539	0.125
6. Education level	0.106	1.434	0.153	0.053	0.867	0.387
7. Total sport experience	0.115	1.330	0.185	0.113	1.590	0.113
8. Current branch experience	−0.081	−0.796	0.427	−0.055	−0.654	0.513
9. Duration with current coach	0.058	0.689	0.492	0.002	0.034	0.973
10. National athlete	0.070	1.132	0.259	0.038	0.750	0.454
**11. Coach-Athlete Relationship (CAR)**	-	-	-	**0.560**	11.297	**<0.001**
F		1.604			13.746	
R^2^		0.056			0.361	
Adjusted R^2^		0.021			0.334	

Standardized beta coefficients are used. Model (*p*) < 0.001; Tolerance = 0.970; VIF = 1.031; Durbin Watson = 1.924.

## 4 Discussion

The main purpose of this study was to examine the relationship between CAR quality and TP, CP, and overall AP. The correlation analysis results showed that CAR was positively and significantly correlated with TP *(r* = *0.412, p*<*0.001)*, CP *(r* = *0.465, p*<*0.001)*, and AP *(r* = *0.461, p*<*0.001)*. Hierarchical regression analysis results showed that CAR had a significant and positive association with TP, CP, and overall AP. Accordingly, CAR was found to be associated with TP *(*β = *0.514)*, CP *(*β = *0.563)*, and AP *(*β = *0.560)*. Therefore, the findings are consistent with H1, H2, and H3. Notably, CAR was associated with CP *(*β = *0.563)*, AP *(*β = *0.560)*, and TP *(*β = *0.514)*.

The literature includes studies consistent with our findings. For example, Akbiyikli and Sürgevil Dalkiliç (2020) found that high-quality CAR is positively associated with elite athletes' TP and CP. In parallel, other studies have demonstrated a positive association between CAR and AP (Duyan, 2021; Pan and Sui, 2025). Zhang and Rhim (2024) demonstrated that CAR had a positive association with motor behavior among Chinese college athletes, with psychological needs as a factor in this relationship. Yuan (2024) found that college athletes generally held favorable perceptions of their CAR, with male athletes reporting more positive relationships than their female counterparts.

A qualitative study on Olympic medal winning athletes showed that CAR quality was associated with athletes' optimal performance and success (Jowett and Cockerill, 2003). Research in team sports also supports this finding; successful volleyball teams reported more positive CAR and higher goal perception compared to less successful ones (Mata and Da Silva Gomes, 2013; Vieira et al., 2015). Similarly, athletes' trust and commitment to their coaches are significantly and positively related to their AP (Nikbin et al., 2014). Overall, these findings suggest that the coach‘s communication style and behaviors (Hanin, 2007), and an appropriate level of closeness (Parry, 2015), are associated with athletes' optimal performance levels. To be associated with athlete performance, it is of great importance to improve the quality of the CAR and to manage this positive climate in a way that meets athletes' expectations.

TP, on the other hand, mainly includes factors such as technical competence, tactical understanding, physical readiness, and individual skills (Mageau and Vallerand, 2003). A positive or high-quality CAR is positively associated with athletes' openness to learning, effort, and competence perception. It is associated with their ability to assimilate instructions and technical feedback from the coach (Davis et al., 2022). For example, through the trust an athlete places in their coach, they can learn challenging technical instructions and show effort to follow training directives (Hampson and Jowett, 2012). This process is associated with technical learning and skill development, thereby being associated with TP (Mageau and Vallerand, 2003). It is noted that coaches' educational approach and decision-making behaviors are associated with athlete satisfaction and performance (Zhu et al., 2024). A recent study on adolescent basketball players found that high-quality CAR was associated with players‘ shooting skill performance throughout the season and was associated with training engagement (Luo et al., 2025). Another study on young basketball players determined that verbal encouragement from the coach during matches was associated with psychophysiological and technical skills, reporting that this was associated with athletes' relationships with their coaches and was associated with game performance (Yilmaz et al., 2025). These findings are consistent with H1.

CP encompasses behaviors such as cooperation, dedication, social support, and extra effort shown by an athlete within the team (Wang and Tong, 2024). It can be said that these behaviors are closely related to social and emotional motivations. According to Self-Determination Theory, the CAR is associated with intrinsic motivation by satisfying the athlete‘s needs for autonomy, competence, and relatedness, thereby being associated with CP (Ryan and Deci, 2000; Mageau and Vallerand, 2003). High-quality CAR are reported to be associated with a sense of belonging, motivation, psychological well being, and social bonds. For instance, Longakit et al. (2024) found that CAR is significantly associated with sports engagement through athletes' intrinsic and extrinsic motivation. Li et al. (2025), in a mixed-methods study on Chinese volleyball players, reported that athlete motivation was significantly associated with individual athlete performance, followed by CAR and team cohesion. In a meta-analysis examining the relationship between CAR quality and young athletes' performance, Bora (2025) concluded that a positive CAR is associated with more favorable young athlete performance. Liu et al. (2025) found that the leadership behaviors displayed by coaches are positively associated with athlete performance. Davis et al. (2018) showed that positive CAR quality is associated with reduced psychophysiological exhaustion in team-sport athletes and more favorable physical and cognitive performance. These findings are consistent with H2.

AP, as a holistic construct encompassing both task and contextual dimensions, was also significantly associated with CAR in this study *(*β = *0.560, p*<*0.001)*. This finding is in line with H3 and consistent with previous research that has conceptualized AP as a unified construct. For instance, Duyan (2021) reported a significant positive association between CAR and performance among combat athletes *(*β = *0.259)*. Moreover, extending this evidence to the relationship between interpersonal dynamics and athlete outcomes, Yildiz (2011) found that low-quality leader-member exchange (LMX) was significantly associated with burnout levels among professional footballer. Given that burnout is known to be negatively associated with AP, these findings are consistent with the relevance of the CAR in relation to performance outcomes. The findings of the present study suggest that CAR may be influential in the Turkish university student-athlete context, where the CAR may be relevant to AP perceptions.

Furthermore, the finding that CAR was associated with CP *(*β = *0.563)* and TP *(*β = *0.514)* is consistent with the role of CAR in relation to athletes' behaviors within the team. This is consistent with the theoretical underpinnings of the 3C model, which focuses on the emotional and relational aspects of the coach-athlete dyad (Jowett and Ntoumanis, 2004). Coaches who build close, committed, and complementary relationships with their athletes are associated with team cohesion, prosocial behaviors, and overall team success. These results can be considered consistent with the literature that shows high-quality CAR -explained by Jowett and Ntoumanis's (2004) 3C model-are associated with fundamental elements such as psychological trust, belonging, and mutual cooperation. Especially, athletes report motivation, feel competent, and focus on their performance through the emotional support and trust they receive from their coach (Jowett, 2017).

The findings of this research demonstrate that CAR quality is significantly and positively associated with TP, CP, and overall AP. The results indicate that a high-quality relationship (a) is associated with TP in relation to technical-tactical development, effort level, and competence perception; (b) is associated with CP in relation to cooperation, voluntary effort, and prosocial behaviors within the team; and (c) is associated with overall AP as a holistic construct, integrating both task and contextual dimensions.

### 4.1 Theoretical and practical implications

#### 4.1.1 Theoretical implications

The findings of this study add to the CAR literature by simultaneously examining the associations of CAR with two performance sub-dimensions (TP and CP) and their composite overall score (AP). By moving beyond traditional approaches that have largely focused on general performance outcomes, often measured through self-report scales (Roux and Trouilloud, 2021), this study examines the differentiated associations of CAR with task-oriented technical skills vs. social-psychological processes within the team. This finding is consistent with how CAR relates to both individual technical development and prosocial team behaviors, rather than treating performance as a unidimensional construct.

Within the framework of SDT (Ryan and Deci, 2000), the findings are consistent with the possibility that CAR is related to athletes‘ intrinsic motivation in the context of basic psychological needs for autonomy, competence, and relatedness, thereby being associated with both TP and CP. This is consistent with the theoretical assumption that positive interpersonal relationships are associated with psychological need satisfaction in sport settings (Mageau and Vallerand, 2003). The finding that CAR was associated with CP *(*β = *0.563)* and TP *(*β = *0.514)* is consistent with the theoretical emphasis on the emotional and relational aspects of the coach-athlete dyad within the 3C model (Jowett and Ntoumanis, 2004). This suggests that the CAR may be important in relation to athletes' social and cooperative behaviors, which in turn are associated with team cohesion and collective efficacy (Jowett and Chaundy, 2004; Hampson and Jowett, 2012).

Moreover, the significant positive association between CAR and overall AP (β = *0.560)* is consistent with the holistic conceptualization of AP as a construct that integrates both task and contextual dimensions. This finding is consistent with theoretical approaches that view AP as a multidimensional phenomenon associated with both technical-tactical skills and psychosocial factors. The hierarchical regression approach employed in this study, which controlled for demographic variables (gender, age, sport type, experience, and national athlete status), allows the association between CAR and performance outcomes to be examined after accounting for individual difference factors. This methodological approach allows for the examination of an association between CAR and performance after accounting for the demographic variables included in the models.

#### 4.1.2 Practical implications

The research results offer evidence-based practical strategies for coaches, sport psychologists, and athletes:

First, the finding that CAR was significantly associated with the self-reported performance dimensions examined in this study suggests that coaches may benefit from prioritizing the development of high-quality relationships with their athletes as a core component of their coaching philosophy, as these relationships were associated with more positive performance perceptions. Coach education programs could consider incorporating modules on interpersonal skills, communication strategies, and relationship management.

Second, given that CAR was associated with CP (cooperation, dedication, social support, and extra effort), coaches may focus on establishing a supportive team environment where athletes feel valued, respected, and psychologically safe. This could involve regular team meetings, encouraging open communication, recognizing prosocial behaviors, and modeling respect and fairness. While this study examined TP, CP, and their composite overall score (AP), it is important to acknowledge that the coach‘s role in athlete development may be associated with other outcomes not captured by these measures, such as physical fitness, tactical awareness, psychological resilience, and long-term athlete development (Côté and Gilbert, 2009; Jowett, 2017).

Third, in line with the findings of Pan and Sui (2025) and Davis et al. (2019), which also are consistent with the positive association between CAR and performance, coaches may consider systematically integrating relationship-building activities into their training periodization. Activities such as goal-setting sessions, individual check-ins, team-building exercises, and open feedback mechanisms may be associated with the coach-athlete bond and may also be associated with more positive self-reported performance perceptions.

Fourth, considering that gender was significantly associated with TP and AP (with beta coefficients negative, indicating differences between male and female athletes), coaches may benefit from adopting gender-sensitive coaching approaches. Tailoring communication styles, motivational strategies, and feedback to the specific needs of male and female athletes may be associated with positive CAR experiences.

Fifth, since the explained variance (R^2^) for performance models ranged from 29.8% to 33.4%, it is important to acknowledge that performance is associated with multiple factors beyond CAR (e.g., physical capacity, technical skills, tactical awareness, environmental factors). Therefore, relationship-building could be integrated as one component of a multi-faceted approach to athlete development, alongside physical conditioning, technical training, and tactical preparation.

Sixth, sports clubs and athletic departments could consider regular assessment of CAR quality using validated instruments such as the CART-Q (Jowett and Ntoumanis, 2004). Such assessments might be associated with awareness of relationship dynamics, although the role of monitoring in performance outcomes requires further empirical investigation.

Seventh, coaches may consider receiving training in communication strategies (motivational, support, and conflict management strategies) as outlined in the COMPASS model (Rhind and Jowett, 2010). Effective communication is relevant to energizing and maintaining quality CAR, and previous research has linked this to performance outcomes.

### 4.2 Limitations and suggestions for future research

Several limitations should be considered when interpreting the findings of this study: (a) First, the cross-sectional nature of data collection makes it difficult to determine causal relationships between variables. Longitudinal studies could more clearly reveal how the CAR is associated with performance over time and how performance evolves. (b) Second, this research focused on direct associations; potential factors such as motivation, self-efficacy, team processes, or burnout were not modeled. Future studies could provide deeper insight by testing these psychological processes. (c) a central limitation of this study is that all performance measures were based on athletes‘ self-reports. Therefore, the findings reflect perceived performance rather than objective performance outcomes (e.g., competition results, coach evaluations, or physical performance tests). While self-reported performance is a valid and widely used construct in sport psychology research (Jowett and Slade, 2021), and correlates positively with objective indicators in many contexts, it is important to recognize that athletes' perceptions may not always align with objective performance measures due to factors such as self-enhancement bias, social desirability, or limited self-awareness. Additionally, the use of self-report measures may introduce common method variance, as all variables were collected from the same source at the same time point. Future studies could incorporate multi-method approaches, including objective performance indicators and coach perspectives, to complement these findings and provide a more comprehensive assessment of the CAR-performance relationship. (d) Finally, the study was conducted within a specific cultural and sporting context. The generalizability of the findings to athletes from different cultures, different sports, or different competitive levels may be limited.

## 5 Conclusion

In conclusion, this study found that the CAR is positively associated with AP. Notably, CAR was associated with CP, which is consistent with the role of positive CAR in relation to athletes' social, cooperative, and team-oriented behaviors. The findings suggest that the quality of the CAR may be an interpersonal factor associated with both task-related and contextual aspects of AP.

From a practical perspective, coaches may benefit from prioritizing effective communication, supportive leadership, and sensitivity to athletes' psychological and interpersonal needs. Developing high-quality CAR may be associated with a supportive team environment and with behaviors that extend beyond athletes' individual TP.

## Data Availability

The raw data supporting the conclusions of this article will be made available by the authors, without undue reservation.
